# Identification of endometrial cancer methylation features using combined methylation analysis methods

**DOI:** 10.1371/journal.pone.0173242

**Published:** 2017-03-09

**Authors:** Michael P. Trimarchi, Pearlly Yan, Joanna Groden, Ralf Bundschuh, Paul J. Goodfellow

**Affiliations:** 1 Department of Cancer Biology & Genetics, The Ohio State University, Columbus, Ohio, United States of America; 2 Department of Internal Medicine, College of Medicine, The Ohio State University, Columbus, Ohio, United States of America; 3 Center for RNA Biology, Department of Physics, Department of Chemistry & Biochemistry, and Department of Internal Medicine, and Center for RNA Biology, The Ohio State University, Columbus, OH, United States of America; 4 Department of Obstetrics and Gynecology, College of Medicine, The Ohio State University, Columbus, Ohio, United States of America; Sapporo Ika Daigaku, JAPAN

## Abstract

**Background:**

DNA methylation is a stable epigenetic mark that is frequently altered in tumors. DNA methylation features are attractive biomarkers for disease states given the stability of DNA methylation in living cells and in biologic specimens typically available for analysis. Widespread accumulation of methylation in regulatory elements in some cancers (specifically the CpG island methylator phenotype, CIMP) can play an important role in tumorigenesis. High resolution assessment of CIMP for the entire genome, however, remains cost prohibitive and requires quantities of DNA not available for many tissue samples of interest. Genome-wide scans of methylation have been undertaken for large numbers of tumors, and higher resolution analyses for a limited number of cancer specimens. Methods for analyzing such large datasets and integrating findings from different studies continue to evolve. An approach for comparison of findings from a genome-wide assessment of the methylated component of tumor DNA and more widely applied methylation scans was developed.

**Methods:**

Methylomes for 76 primary endometrial cancer and 12 normal endometrial samples were generated using methylated fragment capture and second generation sequencing, MethylCap-seq. Publically available Infinium HumanMethylation 450 data from The Cancer Genome Atlas (TCGA) were compared to MethylCap-seq data.

**Results:**

Analysis of methylation in promoter CpG islands (CGIs) identified a subset of tumors with a methylator phenotype. We used a two-stage approach to develop a 13-region methylation signature associated with a “hypermethylator state.” High level methylation for the 13-region methylation signatures was associated with mismatch repair deficiency, high mutation rate, and low somatic copy number alteration in the TCGA test set. In addition, the signature devised showed good agreement with previously described methylation clusters devised by TCGA.

**Conclusion:**

We identified a methylation signature for a “hypermethylator phenotype” in endometrial cancer and developed methods that may prove useful for identifying extreme methylation phenotypes in other cancers.

## Introduction

Cancers develop and progress as a result of accumulation of mutations that alter the coding sequence of genes, as well as changes in gene expression. Changes in gene expression in cancers are associated with alteration in transcription factors, mutations in DNA binding elements, miRNAs, and chromatin remodeling. Chromatin remodeling, including epigenetic modifications to histones and DNA methylation, normally plays a key role in cell differentiation; stably switching cellular pathways on/off until the cells reach a terminally differentiated state that is typically irreversible. Epigenetic changes can lead to tumor suppressor silencing or re-expression of oncogenes in tumor cells, contributing to dysregulation of genes and pathways important in tumorigenesis [1].

DNA methylation is one of the better understood mechanisms of epigenetic control. DNA methylation in humans is mediated by the DNA methyltransferases DNMT1 and DNMT3, which add a methyl group to the 5’ carbon of cytosine. In differentiated cells, DNA methylation occurs in the context of cytosine followed by guanine (CpG) in the DNA sequence. DNA methylation in promoter CpG islands (CGIs) has been shown to mediate stable gene silencing. Tumor suppressor silencing associated with DNA methylation is found in a wide range of tumor types [2]. It is not surprising that DNA methylation is recognized as a potential biomarker [3]. Methods that can be linked to DNA sequencing have been developed to assess methylation, including affinity-based capture of methylated regions and bisulfite conversion [4].

The CpG island methylator phenotype (CIMP) is a cancer-specific accumulation of DNA methylation in CGIs. Originally identified in colorectal cancer [5], CIMP has since been identified in multiple cancer types: glioma [6], breast cancer [7], acute myeloid leukemia [8], gastric cancer [9], clear cell renal cell carcinoma [10], oral squamous cell carcinoma [11], hindbrain ependymomas [12] and endometrial cancer (EC) [13,14]. CIMP arises early in tumorigenesis as evidenced in some colorectal serrated adenomas prior to malignant progression and the development of microsatellite instability (MSI) [15,16]. It can lead to multiple changes in gene expression and, with that, altered tumor biology. CIMP is associated with good prognosis in some cancer types (e.g., colorectal, breast) and poor prognosis in others (e.g., renal cell carcinoma) [17]. In addition to potential roles as a biomarker for precancerous lesions and prognosis in established tumor, CIMP could also represent a therapeutic target for demethylating therapies [18]. Despite its potential diagnostic, prognostic and therapeutic value, CIMP and its manifestations in different cancer types remain poorly understood.

Defining CIMP at the genome level requires extensive methylome profiling. Methylome profiling in ECs has been completed largely through sampling small numbers of CpGs, either at candidate regions or using more general methods in a modest number of tumors. Several methods have emerged to profile the methylome, including the Infinium beadchip [19] and affinity-based methylation capture followed by shotgun sequencing (e.g., MethylCap-seq [20]). The Infinium beadchip has been a method of choice for analyzing tumor DNAs because it is cost-effective, scalable, has demonstrated high accuracy and reproducibility, and has a user-friendly analysis pipeline. The method relies on hybridization of bisulfite-converted DNA to the beadchip, followed by single-base extension. The end result is a readout of percent methylation for individual CpGs, with ~7 CpGs assessed per promoter CGI using the HumanMethylation 450 platform. At the genome level, approximately 8% of the CpGs in promoter CGIs are evaluated. The methylation status of CpGs near those directly assessed is assumed to be similar. MethylCap-seq is one of several affinity-based capture methods that leverage shotgun sequencing to assess methylation patterns. MethylCap-seq uses the MBD2 protein to capture methylated fragments, which are then sequenced to yield piles of methylation tags across the genome [21]. By comparing tag frequency between samples, relative methylation levels can be inferred for a given region. As sequencing costs continue to fall, MethylCap-seq and similar methods will become increasingly cost-effective. For analysis of promoter CGI, MethylCap-seq has a particular advantage over Infinium: average methylation over the regions is measured, rather than assumed.

The purpose of this study was to develop a signature for methylation in endometrial cancers that distinguishes tumor from normal endometrium, and that has potential to classify tumors as having discrete levels of DNA methylation. We developed a 13-region signature that stratified endometrioid endometrial tumors based on CGI methylation status. The signature distinguishes tumors from both normal controls and adjacent normal tissue. This signature was based on a training set of MethylCap-seq data and validated using TCGA Infinium datasets. This signature could prove useful for detecting and classifying endometrioid endometrial carcinomas.

## Materials and methods

### Patient samples and sequence data

Seventy-six primary human endometrioid endometrial cancer and 12 nonmalignant endometrial samples were analyzed from a previously published cohort [22]. The normal endometrial tissues were from patients who did not have endometrial cancer and are thus referred to as “unmatched”. Cohort characteristics are shown in S1 Table. A sequencing read summary is provided in S2 Table. All studies involving human endometrial cancer samples were approved by the Human Studies Committee at the Washington University and at The Ohio State University.

### MethylCap-seq quality control

MethylCap-seq quality control was implemented as previously described [23]. Fourteen of 102 samples showed evidence of poor methylated fragment enrichment or poor sequencing reproducibility and were excluded from analysis, leaving 76 tumors and 12 normals. This method was demonstrated to reduce noise in methylation signal and improve the ability to discriminate between tumors and normal tissue.

### MethylCap-seq data analysis

Sequence files were aligned and processed as previously described [23]. Reads were extended to the average fragment length and the resulting count distribution was normalized against the total aligned reads by conversion to reads per million (RPM). Differentially methylated promoter CGIs were identified by performing a Wilcoxon rank sum test for each CGI across the two sample groups being considered. Results were adjusted for multiple comparisons by setting a false discovery rate (FDR) cutoff of 0.05. Methylation was categorized by genomic feature as follows: CpG islands (CGI, as defined in the UCSC genome browser), promoters (2kb in length, 1kb upstream and downstream of the transcription start site (TSS)), CGI shores (200bp to 2kb distant from both ends of each CGI), and the first exon of RefSeq genes. CGIs were further subdivided by proximity to promoters (within 10kb upstream or 1kb downstream of a 2kb promoter), and 2kb promoters were subdivided by overlap with CGI.

### Infinium validation of methylation signature candidates

Eleven of 76 tumors were chosen for technical validation (S1 Table) using the Infinium HumanMethylation 450 beadchip platform, a well-validated bisulfite-based method for assessing methylation of individual CpGs genome-wide. The assay was performed according to manufacturer protocol by the University of Southern California Epigenome Center. Methylation was reported using beta-values, a number which represents the fraction of DNA fragments that were methylated at a given CpG site.

### Computation of methylation score using the 13-promoter CGI signature

Methylation score was computed by taking the average of the beta-values for all probes within a promoter CGI, then averaging the result across the 13-promoter CGI in the signature. The final signature comprised a total of 88 Infinium HumanMethylation 450 probes.

### *In silico* analysis of TCGA endometrioid endometrial tumors

Methylation was analyzed for 203 endometrioid endometrial tumors from the original published TCGA cohort of 373 endometrial tumors [24]. For 170 tumors, Infinium HumanMethylation 450 data were lacking. Non-endometrioid endometrial cancers were not analyzed. Some analyses assessed fewer than 203 samples due to gaps in data availability for each assay. Methylation was assessed using Level 3 data from The Cancer Genome Atlas Data Portal, while clinical and molecular correlating data were gathered from cBioPortal for Cancer Genomics (Memorial Sloan Kettering Cancer Center).

### Replicate signature analysis

To demonstrate the reproducibility of our method for identifying tumors with a CpG island methylator phenotype, two additional 13-region signatures were compiled from the original list of top differentially methylated promoter CGIs between CG island highly methylated (CGI-H) and CG island low level methylation (CGI-L) tumors in the initial MethylCap-seq analysis. CGI-L tumors in the discovery set were defined as showing promoter CGI methylation signal of less than 5000 RPM, while CGI-H tumors were defined as showing signal greater than 15000 RPM (three-fold difference in signal). Normal controls showed an average methylation signal of 4771 RPM and a maximum methylation signal of 8261 RPM. These definitions were intended to capture the most extreme methylation phenotypes for subsequent analysis, rather than include all tumors with aberrant methylation patterns. For this replicate signature analysis, regions that had already been considered for the original signature were excluded from this analysis. Mirroring the technical validation of the original signature, candidate regions that showed <0.1 difference in average beta-value between groups in the Infinium technical validation set were discarded. An additional negative control signature was populated with the 13-promoter CGI that showed the least differences in methylation between groups in the discovery set (as determined by fold change). Endometrioid endometrial tumors from the test set were indexed using all four signatures, and methylation score was computed using the average beta-value of the regions in each signature. Rank correlation of tumor methylation scores between replicate signatures and the original signature was compared using a Spearman test.

## Results

### Characterizing a CpG island methylator phenotype

**Fig 1:**
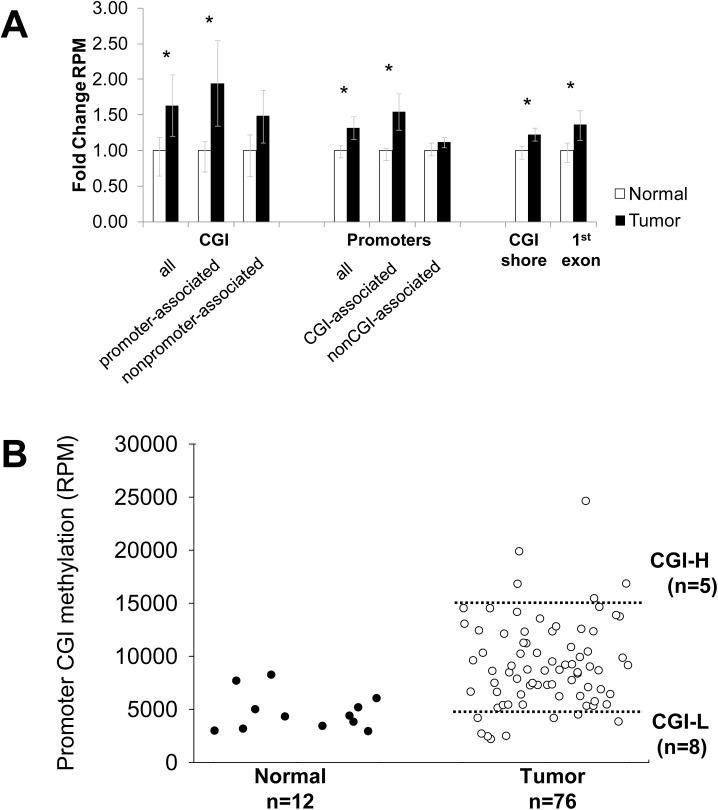
Endometrioid endometrial cancers show increased methylation in promoter CGIs. **(A)** MethylCap-seq normalized signal compared between tumors (N = 76) and normals (N = 12) and plotted across autosomes for three genomic features: CpG islands (CGI), promoters (1kb upstream and downstream of the TSS), CGI shores (200–2000bp 5’ and 3’ of CGIs), and the first exon of RefSeq genes. CGIs were further classified as “promoter-associated” (<10kb 5’ or 1kb 3’ of the transcription start site) or as nonpromoter-associated. Methylation levels were also compared based on the presence or overlap with a CGI within the 2kb promoter. Bars denote mean fold change relative to normal controls; error bars mark 25th and 75th percentiles. Asterisks denote Bonferroni-adjusted Wilcoxon rank sum test p<0.05. **(B)** Pattern of promoter CGI methylation in normal and tumor tissues. Most endometrioid endometrial cancers show increased promoter CGI methylation compared to normal controls, with a subset of highly methylated tumors (CGI-H) showing over 3-fold more methylation compared to tumors with low level methylation (CGI-L). Thresholds were drawn at the upper and lower extremes of the tumor methylation spectrum (dotted lines) to define CGI-H and CGI-L specimens.

Methylome data from a previously reported MethylCap-seq study of 76 endometrioid endometrial carcinomas and 12 normal endometrial tissue controls [25] were analyzed (S1 Table). Patterns of methylation in the tumors and normal DNAs were compared [26,27]. Normal tissues had low level methylation compared to tumors with much less variability in overall methylation than was seen in tumors. Overall, cancers showed a nearly 2-fold increase in methylation of promoter CGIs, with less pronounced gains in methylation of CGI shores (Fig 1A). The increased methylation in genic regions was greatest at the promoters, but was also seen in first exons. Overall, promoter CGI methylation was highly variable with the greatest variation seen in tumor DNAs (Fig 1B). CGI tumor methylation ranged from slightly below the levels seen in normal endometrial tissues to 5-fold higher than normals. Among the 76 tumors investigated, five stood out as having distinctly higher levels of CGI methylation (referred to as CGI-H for highly methylated) and a number of tumors had methylation levels comparable to that seen in the normal endometrial tissues (CGI-L for low level methylation)(S1 Table). To determine if different efficiencies in the methylated fragment enrichment (rather than biological differences in methylation) might explain the variation in methylation seen across the DNAs investigated, we compared levels of nuclear CGI and mitochondrial DNA methylation. A positive correlation could indicate sample-specific differences in efficiency of capture of methylated DNA. Nuclear and mitochondrial methylation levels were not correlated (Spearman r = -0.15, p = 0.2, data not shown). Given the fact that nuclear and mitochondrial methylation are mediated by different processes in distinct cellular compartments [28,29], we reasoned that the lack of correlation made it unlikely that differences in overall methylation were attributable to technical differences/enrichment bias.

**Fig 2:**
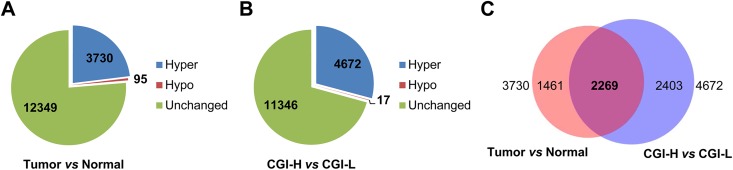
CGIs methylated in CGI-H tumors account for many of the gains that distinguish endometrial cancer and normal endometrial DNAs. **(A)** Endometrioid cancers show increased methylation of >20% of promoter CGI compared to unmatched normal controls (normal endometrium from noncancer patients). Number of loci that were hypermethylated (Hyper), hypomethylated (Hypo), or unchanged in cancers relative to normal are shown. **(B)** CGI-H tumors have increased methylation at nearly 30% of promoter CGI compared to tumors with methylation similar to normal controls (CGI-L tumors).**(C)** Extensive overlap in the hypermethylated regions that distinguish tumor and normal and CGI-H and CGI-L tumors.

Comparison of promoter CGI methylation in normal and tumor tissues revealed an overall increase in methylation in tumors, consistent with a CIMP (Fig 2A). The differences in CGI methylation between the most highly methylated (5 CGI-H tumors) and least methylated tumors (8 CGI-L tumors) were, as expected, almost all gains (4,672 hypermethylated *vs* 17 hypomethylated) (Fig 2B). The extensive variability in CGI methylation involved 29% of all promoter CGIs. Among the 4,672 CGIs hypermethylated in the CGI-H tumors, 2,269 (49%) overlapped with the hypermethylated CGIs for the tumor *vs* normal comparison (Fig 2C). The overlap is more than twice than expected (49% *vs* 23%, Chi squared p-value < 0.001). The loci in the overlap presumably include “hotspots” in the genome that are likely to acquire methylation in endometrial tumorigenesis. Pathway analysis of these 2,269 shared hypermethylated promoter CGIs showed enrichment for known targets of epigenetic regulation, including targets of the Polycomb Repressor Complex and regions known to be methylated in other cancers (S3 Table).

### Technical validation of highly methylated CGIs and development of an endometrial cancer methylation signature

**Fig 3:**
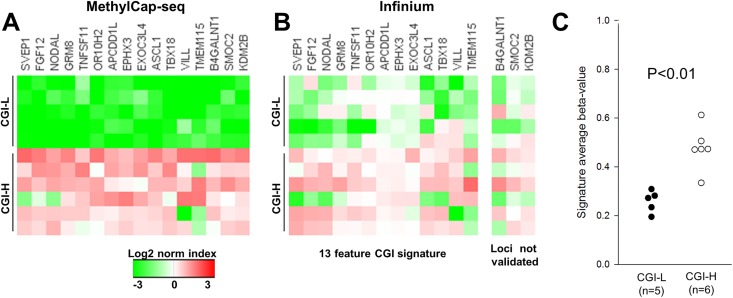
Validation of methylation differences and identification of a 13-promoter-associated CGI panel that distinguish tumors with high and low level promoter CGI methylation. Technical validation for 11 of the 76 tumors initially analyzed using MethylCap-seq was undertaken using the Infinium beadchip. **(A)** Methylation patterns for CGI-H (N = 6) and CGI-L tumors (N = 5) for the 16 promoter-associated CGI signature candidates using MethylCap-seq (Table 1). Relative methylation was compared between regions by normalizing the region average, then applying a log2 transformation. **(B)** Methylation levels for the same 11 tumors based on Infinium HumanMethylation 450 analysis. Tumors were indexed using the average beta-value of all probes in each region (total of 88 probes). Two candidate regions that showed <0.1 difference in beta-value and p>0.05 between the high and low groups and one additional region that showed a negative difference in beta-value were considered not validated (*B4GALNT1*, *SMOC2* and *KDN2B*). **(C)** A methylation score (average of the beta-values of the 13-validated promoter CGIs) plotted for each tumor. P <0.01 for the two groups (Student’s t-test).

The 16 CGIs showing the most significant or largest fold differences between CGI-H and CGI-L tumors, and that had distinguished tumor and normal DNAs, were considered candidates for a “highly methylated” signature for ECs. The number of CpGs in the 16 CGIs ranged from 23 to 234, with the MethylCap fold enrichment ranging from 11.7X to 18.9X (Table 1). When the methylation levels of the 16 CGIs were compared in the CGI-H and CGI-L tumors (5 and 8 cases respectively) 15 of 16 candidates were, as expected, more methylated in the CGI-H tumors. The exception was the *TMEM115* CGI that was hypermethylated in only 3 of the CGI-H tumors (Fig 3A).

**Table 1:** Promoter CGIs that distinguish CGI-H from CGI-L tumors as measured by MethylCap-seq.

Gene symbol	Chromosomal location	CpG	Fold change
	(hg19)	count	RPM
***TMEM115***	chr3:50402103–50402942	66	18.9
***TBX18***	chr6:85472702–85474132	129	18.9
***NODAL***	chr10:72200065–72201368	106	16.9
***SVEP1***	chr9:113341213–113342029	99	16.2
***TNFSF11***	chr13:43148277–43149282	83	16
***OR10H2***	chr19:15833733–15833983	23	14.5
***KDM2B***	chr12:122016170–122017693	125	14.4
***FGF12***	chr3:192125818–192127991	176	14
***APCDD1L***	chr20:57089460–57090237	71	13.6
***EPHX3***	chr19:15344091–15344419	33	13
***ASCL1***	chr12:103351579–103352695	105	13
***EXOC3L4***	chr14:103557606–103558235	63	12.6
***SMOC2***	chr6:168841818–168843100	125	12.5
***B4GALNT1***	chr12:58025661–58027056	124	12.2
***GRM8***	chr7:126891300–126894205	234	12.1
***VILL***	chr3:38035701–38036000	29	11.7

The 2269 promoter CGI were sorted by Kruskal-Wallis p-value and fold difference for the CGI-H vs. CGI-L comparison; potential candidates were excluded that overlapped regions associated with copy number amplification in TCGA endometrioid samples (8 of the top 50). In addition, a threshold Student p-value of p<0.01 (not corrected for multiple comparisons) was imposed to exclude candidates with large fold differences that appeared to be driven by outlier samples (5 of the top 21).

Analysis of CGI-H and CGI-L tumors using the Infinium HumanMethylation 450 beadchip validated the observed increase in methylation seen with MethylCap for 13 of 16 candidates (Fig 3B). Because DNA was not available for some of the tumors studied by MethylCap (four tumors for which DNA stocks were depleted), the orthogonal validation included only nine of the previously studied cases (4 CGI-H and 5 CGI-L)(S1 Table). One additional case with methylation near the CGI-H cut-off and three additional tumors with methylation close to the CGI-L cut-off (Fig 1B) were analyzed. Candidate hypermethylated loci were considered to be technically validated using the following criteria: beta-value difference of greater than +0.1 (CGI-H–CGI-L) *or* Student’s t-test p<0.1. We purposely set low beta and permissive p values to avoid over-fitting. The 13 validated signature regions comprise a total of 88 Infinium HumanMethylation 450 probes located within the respective CGIs, with a median of six probes per region and a range of 2 to 14 (Table 2).

**Table 2:** Promoter CGIs validated by Infinium beadchip analysis.

Gene symbol	# probes	Δ beta-value^1^	p-value^2^
***SVEP1***	4	0.34	0.01
***FGF12***	14	0.32	0.02
***NODAL***	6	0.31	0.02
***TNFSF11***	10	0.31	0.01
***TBX18***	10	0.27	0.06
***OR10H2***	3	0.26	0.05
***VILL***	2	0.25	0.14
***ASCL1***	9	0.2	0.13
***EPHX3***	3	0.17	< .01
***GRM8***	11	0.15	0.06
***TMEM115***	3	0.12	0.12
***APCDD1L***	9	0.1	< .01
***EXOC3L4***	4	0.04	0.08

^1^ Measured as the average beta-value for CGI-H minus CGI-L tumors

^2^ Calculated using Student’s t-test

The validated signature regions robustly distinguished 5 out of 6 of the CGI-H tumors from the CGI-L tumors (Fig 3B). The aggregate signature composed of these 13 CGI promoter regions likewise distinguished CGI-H from CGI-L tumors (mean average beta-value of 0.47 *vs* 0.26, Student’s t-test p<0.05) (Fig 3C).

### Methylation signature stratifies endometrioid endometrial tumors by methylation phenotype and distinguishes tumors from normal controls in the endometrial cancer TCGA dataset

To test whether the 13-CGI signature that we developed can distinguish methylation phenotypes in an independent cohort, we examined the methylation profiles for endometrioid endometrial carcinomas from TCGA. The available data for 203 ECs generated using the Infinium HumanMethylation 450 beadchip were analyzed [24]. TCGA over-sampled for high grade endometrioid cases with approximately one-third of cases being grade 1, one-third grade 2 and one-third grade 3. The cohort is otherwise largely representative of women with ECs [24].

**Fig 4:**
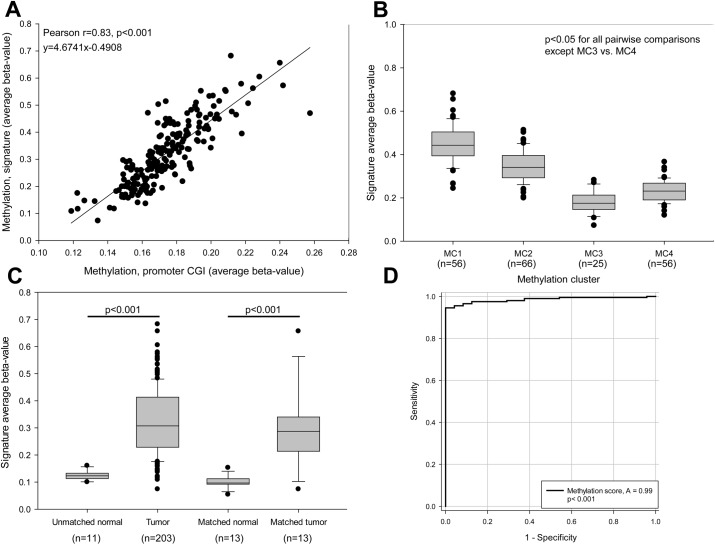
MethylCap-seq 13-feature methylation signature stratifies endometrioid endometrial tumors by methylation phenotype and distinguishes tumors from normal controls. **(A)** Thirteen feature methylation signature score shows a strong linear relationship with overall promoter CGI methylation. Infinium methylation data were analyzed for 203 endometrioid endometrial tumors from TCGA [24]. Average methylation for the 13-signature promoter CGI from Fig 3 were compared against average methylation for all promoter CGI. **(B)** Methylation score distinguishes the TCGA methylation phenotypes. Tumors were grouped into the methylation clusters previously identified in TCGA study and their signature average beta-values plotted as a standard box plot. Signature score distinguished all clusters except MC3 vs. MC4 (p<0.05 for all pairwise comparisons except MC3 vs. MC4 using a Kruskal-Wallis test with Dunn's post-hoc). Whiskers denote 10th and 90th percentiles. **(C)** Methylation signature distinguishes tumors from normal controls. Methylation score was plotted for all tumors in 11 unmatched normal controls (left), as well for tissue from matched tumor and adjacent normal samples (TCGA data). A Wilcoxon rank sum test was used to compare unmatched normals (normal endometrial tissues from women without endometrial cancer) and tumors, while a paired Student’s t-test was used to compare matched normals and matched tumors. **(D)** Thirteen-feature methylation signature distinguishes tumors from normal controls with high sensitivity and specificity. Matched and unmatched normals (see panel C) were pooled, and an ROC curve was generated. Sensitivity represents the true positive rate for tumors at a given signature score threshold (% tumors correctly categorized as tumors), while specificity represents the false positive rate (% normal controls incorrectly categorized as tumors).

Because the 13-CGI methylation signature we devised came from comparison of individual CGIs in tumors that showed the largest differences in overall promoter CGI methylation, we first assessed the relationship between our 13-CGI signature with overall CGI methylation in the TCGA cohort. Our 13-gene signature and overall CGI methylation proved to be highly correlated (Fig 4A) as would be expected for a marker for genome-wide CIMP. Our 13-locus signature was also highly correlated with methylation clusters (MC1-4) developed by TCGA (Fig 4B). The 13-CGI signature scores (beta values) were significantly different for tumors assigned to TCGA MC1 and MC2 clusters (very highly and highly methylated groups) compared to the other two groups (MC3 with methylation comparable to levels seen in normal and MC4 with intermediate methylation). ANOVA revealed that the 13-gene signature scores were significantly different across the four groups. The MC3/MC4 comparison however, showed these two groups have indistinguishable scores/beta values (Fig 4B).

Methylation scores for tumors were compared to the scores for matched and unmatched normal control tissues (Fig 4C). Ninety-five percent (192 of 203) of endometrioid ECs showed a higher methylation score than unmatched normal controls (N = 11), suggesting that overall the 13-region signature could reliably distinguish tumor and normal tissues. Likewise, for the small number of cases with matched normal and tumor tissues (N = 13), tumors had an average 3-fold increase in methylation compared to normal, and only one tumor had methylation levels in the same range as normal tissues. The 13-CGI methylation signature showed a sensitivity of 0.95 +/- 0.03 and a specificity of 0.93 +/- .07 (95% confidence interval) for distinguishing tumors from normal tissue at a methylation score threshold of 0.16 (Fig 4D).

To determine if methylation levels for the 13 CGIs in our signature were related to gene expression, we evaluated the transcript levels using the publicly available RNA-seq data. Seven of the 13 genes showed a significant association between methylation and transcript levels: increased methylation was associated with reduced levels of transcripts (Table 3). *EPHX3* expression decreased notably with increasing promoter methylation (S1 Fig).

**Table 3:** Correlation between promoter CGI methylation and gene expression in TCGA tumors.

Gene symbol	Spearman r	p-value
***EPHX3***	-0.601	2.0 x 10^−7^
***TBX18***	-0.494	2.0 x 10^−7^
***TNFSF11***	-0.379	3.5 x 10^−7^
***VILL***	-0.37	6.7 x 10^−7^
***FGF12***	-0.365	9.7 x 10^−7^
***ASCL1***	-0.243	< .0.01
***APCDD1L***	-0.238	<0.01
***SVEP1***	-0.118	0.12
***NODAL***	-0.113	0.14
***GRM8***	-0.108	0.16
***EXOC3L4***	-0.0245	0.75
***TMEM115***	0.0192	0.8
***OR10H2***	0.164	0.03

### High methylation score is associated with mismatch repair deficiency, high mutation rate, and low somatic copy number alteration

**Fig 5:**
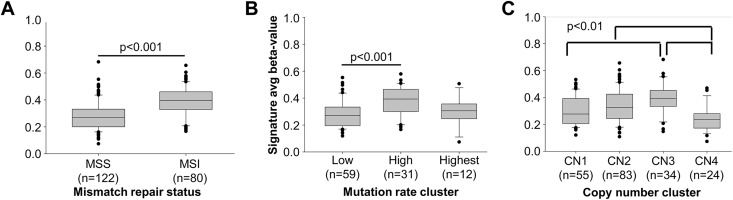
High methylation score is associated with MMR deficiency, high mutation rate, and low somatic copy number alteration. Methylation score was compared among published clusters for 203 endometrioid endometrial tumors in The Cancer Genome Atlas. **(A)** Microsatellite instability status comparing MSS and MSI-positive tumors; **(B)** Mutation rate cluster; **(C)** Copy number (CN) clusters. Statistical comparisons were performed using Wilcoxon rank-sum, ANOVA and Kruskal-Wallis tests.

In colorectal cancer, CIMP is a feature of tumors with defective DNA mismatch repair (MMR) [15]. Tumors with DNA MMR defects have elevated mutation levels and have characteristically accumulated large numbers of strand-slippage mutations that give rise to the MSI phenotype. MMR defects are frequently seen in EC. Epigenetic silencing of the *MLH1* MMR gene associated with hypermethylation in its promoter, accounts for the vast majority of MSI-positive/MMR deficient ECs [30–32]. When we compared our 13-CGI methylation scores for TCGA tumors stratified based on MSI status, mutation rate cluster and copy number cluster, clear differences with all three features were evident (Fig 5). Methylation score correlated with MSI status (MSI+ *vs* microsatellite stable (MSS)), median 0.40 vs. 0.27, Wilcoxon rank sum p<0.001, Fig 5A) and mutation frequency (High *vs* Low clusters, mean 0.38 *vs* 0.28, ANOVA with Holm-Sidak post-hoc p<0.001, Fig 5B). Methylation score also varied with somatic copy number alteration (SCNA) cluster. Clusters 2 and 3 have higher methylation than SCNA cluster 4 (median 0.33 and 0.39 vs. 0.24, Kruskal-Wallis with Bonferroni-corrected Student's t-test post-hoc p<0.01). SCNA cluster 3 also have significantly higher methylation than very low SCNA cluster 1 (median 0.39 vs. 0.28) with cluster 2 having an intermediate value (Fig 5C). Given the highly significant association between MSI and methylation of the MLH1 promoter in sporadic endometrioid endometrial cancers [32], the strong correlation observed between methylation score and MSI was expected. The high mutation TCGA group is greatly enriched for MSI-positive tumors and thus our 13-CGI signature was similarly higher is these tumors. The inverse relationship between methylation score with low SCNA suggests that CIMP/MSI and chromosomal instability may be features of two distinct pathways of tumorigenesis [24].

When we compared our 13-feature CGI methylation score across other published endometrioid endometrial TCGA cancer cluster data (mRNA and miRNA expression) and with clinicopathologic and demographic variables (BMI, stage, grade and relapse-free survival), no significant relationships with methylation score were seen (threshold of p<0.01, data not shown).

### Methodological validity

The reproducibility of the methods used to generate the 13-CGI methylation signature was tested by generating two additional “replicate signatures”, each including 13 different promoter CGI selected using similar methods. As shown in S2 Fig, when the “replicate signatures” were applied to TCGA set, they performed similarly (r = 0.82,0.89 for replicates R1 and R2 *vs* original signature, p<0.001; r = 0.144 for negative control *vs* original signature, p>0.01). This suggests our approach to developing a methylation score is robust. The relationships with molecular signatures associated with our 13 CGI score (MSI, mutation rate, copy number alteration) were evaluated using the “replicate signatures”, revealing similar strong correlations.

### Relationship between a reduced feature CIMP signature and TCGA methylation clusters

We assessed the relationship between the 13-feature CGI signature developed using MethylCap and TCGA methylation clusters [24]. The 13-feature CGI signature (Fig 3B) captures most of the most highly methylated TCGA tumors (those assigned to methylation cluster 1, MC1) with a threshold beta-value of 0.4. All tumors assigned to methylation cluster 3 and 4 (25 and 56 tumors, respectively) have beta-values <0.4 (Fig 4B). The ≥0.4 value, which we consider a marker for high CIMP (CIMP-H), also excludes one of MethylCap-seq CGI-H tumors we profiled with the Infinium HumanMethylation 450 platform (Fig 3C). When the 0.4 beta-value is applied to the 203 TCGA endometrioid ECs, 58 (29%) would be classified as CIMP-H. The size of the group is comparable to the size of MC1 set (56 of 203, 28%).

## Discussion

Aberrant DNA methylation is a feature of most cancers and can be an early event in tumorigenesis [33–36]. There is tremendous variability in both the extent and patterns of methylation across and within cancer types, and profiling methylation has increasingly become part of the molecular phenotyping for tumors. DNA methylation is an attractive biomarker with potential diagnostic, prognostic, and therapeutic applications [3,18,37–39].

Although a CIMP has been defined in a variety of tumor types, there are a limited number of studies that have leveraged genome-wide methylome profiling techniques to examine CIMP in endometrial cancer [24,40,41]. We combined measurement of methylation over CGI regions (MethylCap-seq) which defines large-scale methylation differences with Infinium data, which relies on a smaller number of data points to generate a signature for global differences in methylation that is based on a small number of features. By doing so, we leveraged the increased CpG coverage of enrichment-based methylation profiling to determine which of the smaller number of features best capture large increases in methylation over a CGI region.

Our analysis was based on the premise that a CIMP could be identified based on aggregate methylation, which has not typically been used to define CIMP markers. We demonstrated that the approach for identifying CIMP based on aggregate methylation shows general agreement with a clustering-based approach (Fig 4B), and furthermore show that the methylation score yielded by our signature reflects aggregate CpG island methylation (Fig 4A). In addition, most tumors show more aggregate CGI methylation than normal controls (Figs 1, 4C and 4D), suggesting that promoter methylation is more prevalent in endometrioid endometrial cancer than previously thought.

The aggregate methylation analysis approach that we took is unlike unsupervised clustering methods used in many genome-wide/global methylation studies. MethylCap-seq methods do not require complex data normalization and correction for batch effects necessary for clustering, but do require rigorous quality assurance to avoid technical bias for poor CpG enrichment (elimination of cases with very low levels of CpG methylation and evaluation of mitochondrial methylation). By excluding tumors with very low CpG methylation (presumed to be poor capture of methylated fractions) from analyses, there is the possibility that we fail to consider samples that do indeed have very low CGI methylation (significantly below that of normal tissue). An obvious implication of the bias towards increased methylation is that should a subset of endometrial cancers have a “CpG island hypomethylator phenotype”, they would likely go undetected. The validity of our approach for identifying CIMP in endometrioid endometrial cancer was best evidenced by the strong correlation between what we measured as aggregate promoter CGI methylation in the TCGA data set, and the previously assigned TCGA methylation clusters (Fig 4A). The TCGA data set was not only for a completely different set of tumors, but also relied on an entirely different platform for measuring methylation.

Methylation in normal tissues is highly tissue-specific, and it is not surprising the tumor-specific methylation abnormalities tend to be related to cell of origin [42]. Given the specificity of methylation in normal tissues, it follows that the markers used to define CIMP vary from one cancer type to another. Broad changes in DNA methylation are shared by many tumor types as are a range of sequence/locus-specific changes, but these general methylation abnormalities are not markers for tissue-type CIMP. CGIs for three genes known to be methylated in other tumor types are part of our 13-region signature for endometrioid endometrial cancer: *EPHX3* (ABHD9), *FGF12*, and *ASCL1*. Methylation of *EPHX3* is seen in primary prostate cancers [43]. Methylation of *FGF12* has been reported in colorectal tumors but not in matched controls [44] as has methylation of *ASCL1* [42]. It is appealing to suggest that *ASCL1* and *FGF12* methylation might have diagnostic potential (ability to discriminate between tumor and normal tissues) in both colorectal and endometrial cancer or reflect similarities in the biology underlying these cancer types.

Our study corroborates and expands on TCGA for endometrial cancer consortium methylation profiling and cluster analysis. The CIMP developed by TCGA was based on Infinium methylation data (Infinium HumanMethylation450 platform) that includes 113,521 probes from CpG islands (average of 7 probes per CGI). These promoter CGI have an average length of 904bp and 84 CpGs per island; therefore the Infinium platform measures methylation of 8% of CpGs in promoter CGI. Although it is widely accepted that methylation of Infinium probes is representative of regional methylation, this may not be the case for all tissues or tumor types. Similarly the small number of probes per region may not reflect the region as a whole in tumors with profound dysregulation genome-wide methylation patterns. Our methylation signature is based on genome-wide promoter CGI data collected using MethylCap-seq and the agreement of our methylation score data with the clusters in the TCGA Consortium study validates their method as well as our own. Our CIMP classification based on the 13 loci has an additional advantage in that it requires measuring methylation of only 82 CpGs relative to the large number of probes from across the genome used for clustering in the TCGA Consortium study. Such a 13-feature signature could easily be formatted for low cost high-throughput analysis. The methylation threshold of 0.4 for identifying endometrial CIMP endometrial tumors we established could be used to dichotomize the methylation state. Our data, however, suggest that CIMP in endometrioid endometrial cancer could be viewed as a continuum rather than as a discrete phenomenon (Figs 1B and 4C). We suggest the score threshold of 0.4 for our 13 features distinguished CIMP-H tumors in TCGA data set for 203 endometrioid tumors, but analysis of additional endometrial cancer methylation data sets is warranted.

It is important to note that the methylation of 13 CpG islands included in our methylation signature are correlated with broad gains in CpG island methylation in both our data and in the TCGA data set. The significance of an assigned methylation score is this underlying correlation, not the methylation of the individual islands. Methylation of an arbitrary set of islands could be useful for diagnosis of cancer and could predict response to treatment, but may not in and of themselves indicate an underlying methylator phenotype.

## Conclusion

In summary, we used two methylome profiling techniques to stratify tumors by overall promoter CGI methylation, identified a signature to reproduce this stratification, and verified that classification of tumors using this signature reproduced known characteristics of CIMP tumors (e.g., the association with MSI). More generally, we demonstrated an approach for translating methylome profiling findings to the Infinium platform, which will become increasingly important as more publicly available methylation datasets become available and the associated clinical data mature. Our analyses suggest that widespread promoter methylation is more prevalent in endometrioid endometrial cancer than previously appreciated, and that promoter methylation could be a useful marker for distinguishing tumors and normal tissue.

## Supporting information

S1 Fig Methylation of *EPHX3* is associated with decreased gene expression. RNA expression *vs* promoter CGI methylation of *EPHX3* was plotted for 172 endometrioid endometrial tumors from TCGA. A linear fit line (r^2^ = 0.4) depicts the inverse relationship between RNA expression and methylation, corresponding to a Spearman correlation coefficient of r = -0.60 and p<0.001. TPM indicates transcripts per million, as calculated by RSEM. Methylation beta-value represents the average methylation of all Infinium probes within the CGI.
(TIF)

S2 Fig Replicate 13-region methylation signatures rank tumors similarly. Comparison of original methylation signature methylation levels with values for replicate signatures in TCGA data. Two hundred and three endometrioid endometrial tumors from TCGA were indexed using the average beta-value of all regions in the signature, and relative index values between replicates were compared by plotting as a normalized log2 transformed heatmap. Samples were ranked by the original signature index (O) for visual comparison. Statistical comparison of rank correlation vs. the original signature was performed using a Spearman test (r = 0.82, 0.89 for replicates and p<0.001; r = 0.14 for NC and p>0.01). R1: replicate signature 1, R2: replicate signature 2, NC: negative control.
(TIF)

S1 Table Cohort characteristics. (XLSX)

S2 Table Sequencing summary. (XLSX)

S3 Table Term enrichment associated with hypermethylated promoter CGI (MSigDB Perturbation). (XLSX)
