# Supplementary figures and images for: Identification of endometrial cancer methylation features using combined methylation analysis methods
Source: PLoS One. 2017 Mar 9;12(3):e0173242. doi: 10.1371/journal.pone.0173242 (PMC5344376; doi:10.1371/journal.pone.0173242)

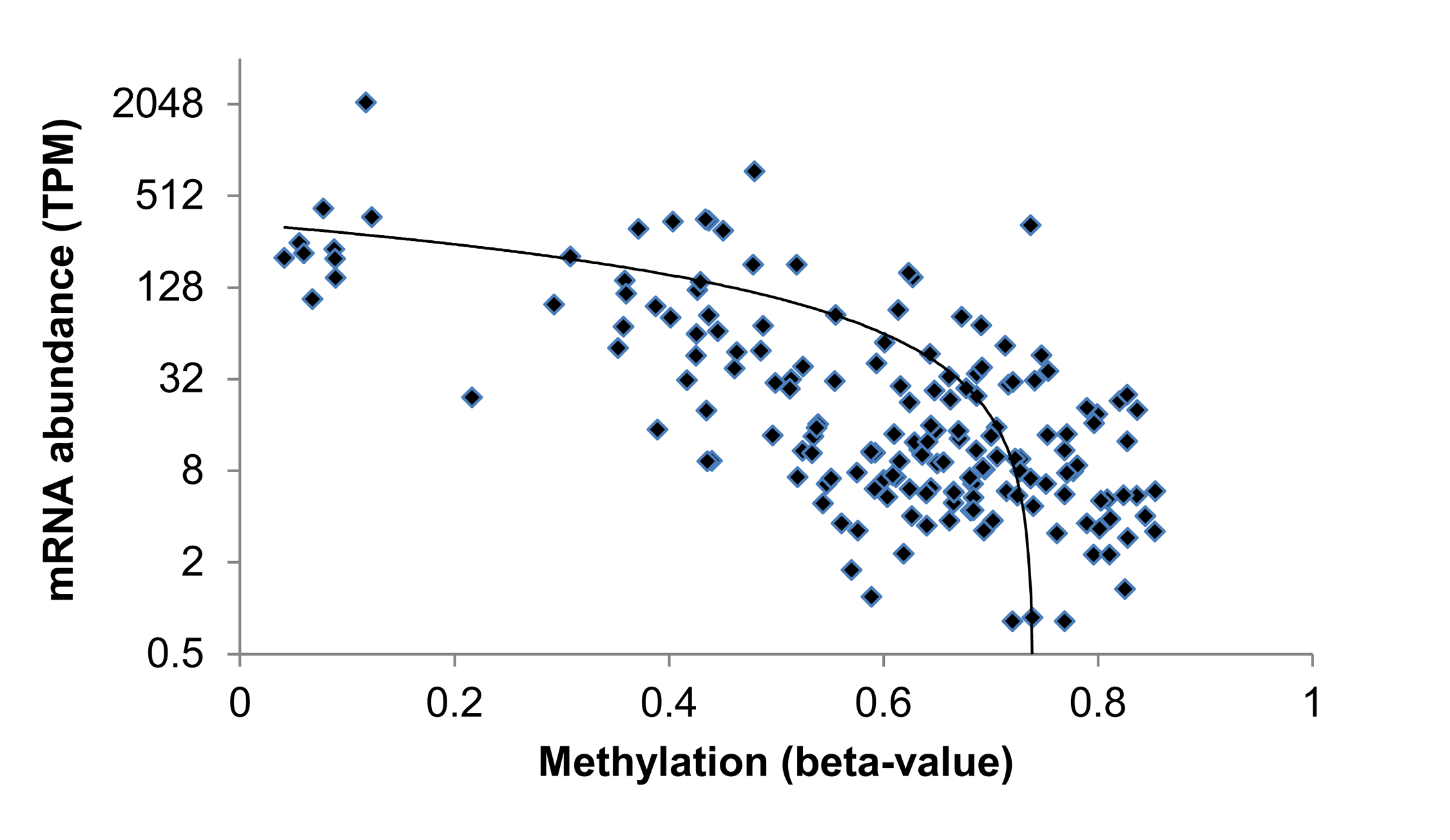

Supplement: S1 Fig — RNA expression vs promoter CGI methylation of EPHX3 was plotted for 172 endometrioid endometrial tumors from TCGA. A linear fit line (r2 = 0.4) depicts the inverse relationship between RNA expression and methylation, corresponding to a Spearman correlation coefficient of r = -0.60 and p<0.001. TPM indicates transcripts per million, as calculated by RSEM. Methylation beta-value represents the average methylation of all Infinium probes within the CGI. (TIF) [file pone.0173242.s001.tif]

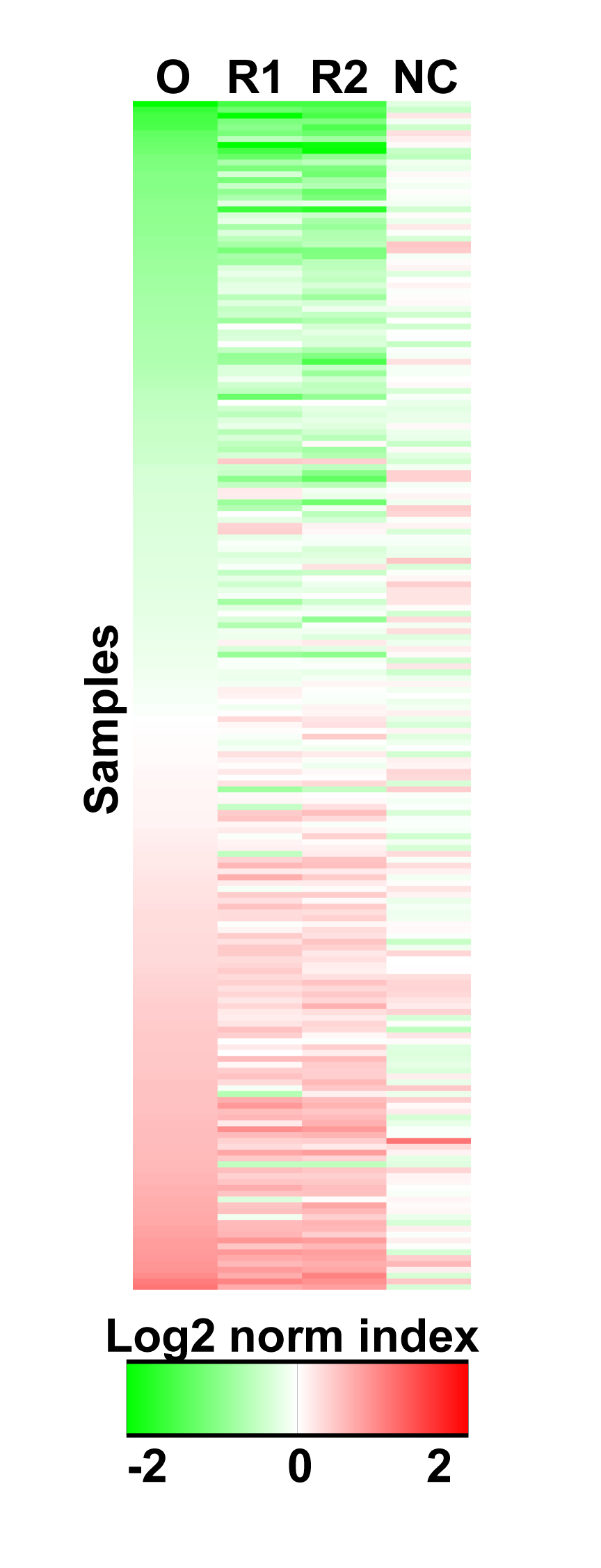

Supplement: S2 Fig — Comparison of original methylation signature methylation levels with values for replicate signatures in TCGA data. Two hundred and three endometrioid endometrial tumors from TCGA were indexed using the average beta-value of all regions in the signature, and relative index values between replicates were compared by plotting as a normalized log2 transformed heatmap. Samples were ranked by the original signature index (O) for visual comparison. Statistical comparison of rank correlation vs. the original signature was performed using a Spearman test (r = 0.82, 0.89 for replicates and p<0.001; r = 0.14 for NC and p>0.01). R1: replicate signature 1, R2: replicate signature 2, NC: negative control. (TIF) [file pone.0173242.s002.tif]
